# Contemporary visualities of ill health: On the social (media) construction of disease regimes

**DOI:** 10.1111/1467-9566.13846

**Published:** 2024-09-20

**Authors:** Stefania Vicari, Hannah Ditchfield, Yuning Chuang

**Affiliations:** ^1^ Department of Sociological Studies The University of Sheffield Sheffield UK

**Keywords:** BRCA, cancer, disease regime, genetic risk, social media, visual methods

## Abstract

First‐person representations of illness have been studied as key to the cultural fabric disrupting dominant practices of ill health or disease regimes. However, the role that digital platforms play in shaping this fabric in contemporary societies has been mostly overlooked. We address this gap by investigating how mainstream social media, as mundane spaces modelled by corporate‐driven techno‐commercial structures, frame specific forms of visuality or ways to see ill health. We reflect on how these forms of visuality relate to existing disease regimes. The article presents an investigation of popular images of BReast CAncer (BRCA) hereditary cancer syndromes posted on Instagram, Twitter (now X) or Facebook over the course of 12 months. By combining cultural analytics, visual network analysis and interpretive techniques, we explore the emergence of platform‐specific visual vernaculars and the visual genres of ill health emerging from these vernaculars. Our analysis suggests that, in the context of BRCA hereditary cancer syndromes, popular social media images primarily exacerbate existing racialised and gendered practices. Where alternative views emerge, in their being shaped by platforms’ attention economies, they often operate in what we define as a ‘liminal space’ of imagination – one that hints at renewed, but not necessarily disruptive and certainly not radical ways to imagine ill health.

## INTRODUCTION

In their 2002 pivotal piece, ‘Seeing health and illness worlds', Harrison lamented the scarcity of visual work in sociological research, recommending in particular that ‘we pay attention to the visual not just as a resource for our studies, but also as a topic in social life’ (868). As a mode of representation dominating the public domain, visual content does ‘social work’: it resonates with existing cultural codes, for instance of gender and colour, reproduces or challenges normative assumptions around what things *should* look like, and offers unique potential to communicate meaning that can hardly be verbalised, like that of chronic pain (Gonzalez‐Polledo & Tarr, 2016) or depression (Hussain, 2020). Ultimately, Harrison stressed the need to consider visual artefacts as discursive devices constructed to account for and represent particular experiences of health and illness.

In this article, we draw attention to visual content shared on mainstream social media platforms. Existing sociological work shows that this content enhances participatory processes related to knowledge sharing and community building (e.g. Cho et al., 2018; Mazanderani, O’Neill, & Powell, 2013). However, the role that digital platforms play in shaping this content has been mostly overlooked. We are interested in the way mainstream social media, as modelled by corporate‐driven techno‐commercial structures, shape specific forms of visuality or ways to see (Rose, 2023, p. 17) ill health. We are asking how these forms of visuality relate to established practices of health and illness, so‐called ‘disease regimes’ (Klawiter, 2004). The article advances a threefold contribution: it brings together medical sociology and critical digital studies to provide insight into the role of the *contemporary* visual in social constructions of health and illness; it develops an innovative approach to study cross‐platform visual content and it proposes a conceptual framework to interpret the role of the contemporary visual vis‐a‐vis dominant societal practices.

In the next part of the article, we will review work on illness and visuality and conceptualisations of technological affordances and platform vernaculars. Then, we will present the methodological design, findings and reflections from a study of popular mainstream social media content about BReast CAncer (BRCA) hereditary cancer syndromes posted on Facebook, Instagram or Twitter (now X) over the course of 12 months. In the concluding section, we will discuss the BRCA visualities emerging from this content and reflect on the broader implications of social media for contemporary practices of ill health.

## BACKGROUND: ILLNESS AS VISUAL

### Visual representations: From methods to topic of research

As methodological resources, visual representations have often been approached because of their *narrative* power. Building on a decade of research into ‘illness narratives' (Hydén, 1997), in 1994, Rich and Chalfen trialled the use of ‘visual illness narratives'. In the context of a research project at Boston’s Children’s Hospital, children and adolescents with asthma were asked to create visual narratives of their illness through filming their lives. The process designed by Rich and Chalfen was meant to give *voice* to those living with the condition, in line with existing (Kleinman, 1988) and later (Frank, 1995) conceptualisations of what illness narratives can add to normalised understandings of health, illness and medicine. The *biographical* dimension emerging in Rich and Chalfen’s (1999) study has also clearly surfaced in the more comprehensive bulk of research employing photovoice (Wang & Burris, 1997) or photo elicitation (Frith & Harcourt, 2007) techniques, where patients are asked to take – and comment on – pictures of their experiences. What, perhaps, is less clear here is the work that images can do in terms of constructing illness in the social world – something that is more clearly addressed when visual representations are investigated as a topic of research.

In fact, narrative, voice and biography also clearly emerge in work more explicitly focused on visual representations as a topic, primarily in studies investigating artefacts that originate outside the research context, like spontaneous first‐person projects, journals or diaries. It is this scholarship that has more openly acknowledged the role played by the medium, for instance, photography, in the forms of representation produced through these artefacts. The medium, in the hands of the patient, ensures some form of ‘control’ (Frith & Harcourt, 2007) over the way illness is made visible, narrated and made sense of. And when it is the patient themselves who initiate these visualisations, the medium is seen as helping to convey additional meaning – a meaning often entrenched with aesthetics and novelty, if not disruption. Breast cancer projects take front stage here. While 1979 Susan Sontag would write of cancer ‘it seems unimaginable to aestheticize the disease’, it is actually in the 1970s that cancer illness experiences entered the public domain. This happened through an aestheticised and subversive representation of the breast cancer experience, namely 1977 photograph ‘The Warrior’ (or ‘Tree Poster’). This shows smiling poet Deena Metzger, her arms outstretched against the sky, bare chested, her right breast missing and the tattoo of a tree branch covering her scar (Henriksen et al., 2011; Radley & Bell, 2007). This photograph suggests a visuality, or a way we are made to see breast cancer, that departs from previous representations of the disease.

‘The Warrior’ has been followed by a number of visual projects that have attracted public attention and sociological inquiry, for instance, British feminist artist Jo Spence’s photographs chronicling her 10‐year experience (1982–1992) of living with breast cancer (Bell, 2002); English model and photographer Matuschka’s 1993 self‐portrait ‘Beauty Out of Damage’, showing her mastectomy scar on the cover of the *New York Times Magazine* (Henriksen et al., 2011; Radley & Bell, 2007); US model and photographer Lynn Kohlman’s (2005) autobiography ‘Lynn Front to Back’ (DeShazer, 2012); American scholar Catherine Lord’s (2004) experimental narrative ‘The Summer of Her Baldness: A Cancer Improvisation’ (DeShazer, 2012); Danish breast cancer survivor’s book montage Sara Bro’s diary (Henriksen et al., 2011) and American artist Martha Hall’s artist books about her breast cancer experience (Radley & Bell, 2007). What suddenly becomes visible in these projects – whether a missing female breast (Matuschka, Metzger), a tattooed scar (Metzger), the emotional distress during or following patient–doctor encounters (Hall, Spencer), a woman dying from cancer (Spencer), the eventfulness of a clinical appointment (Bro, Hall) – challenges traditional western takes on both the (female) body and the hierarchy of relevance in patient–doctor relationships and expertise. As Radley pointed out, these accounts ‘are more than narratives – in the sense of storied tellings – inasmuch as they are exemplars of a way of being in the world’ (1999, p. 791). They represent the embodiment of specific ideological and social practices (Radley, 1993).

Sociologists have pointed at the role of visual representations in offering ‘anchoring potential’ (Radley & Bell, 2007, p. 369) for new social practices, namely in providing material and discursive space that helps establish the significance of these practices. In fact, their acceptance requires ‘the visible, public enactment of new patterns so that “everyone can see” that everyone else has seen that things have changed’ (Swidler, 2001, p. 96). In the context of illness experiences, this anchoring potential contributes to building material and discursive space for shifts in what Klawiter defined as ‘disease regimes': ‘the institutionalised practices, authoritative discourses, social relations, collective identities, emotional vocabularies, visual images, public policies and regulatory actions through which diseases are socially constituted and experienced’ (2004, p. 851). Visual representations construct visualities that feed into the ‘mythology’ (Sontag, 2001[1979]) of a disease, the collection of discourses and symbols commonly accepted to think and communicate about a disease. A shift in this mythology signifies a shift in the overall regime. Between the 1970s and the 1990s, for instance, the visual projects listed above, among others, signified a shift in the breast cancer regime, one that saw changing and expanding practices, especially in relation to gender, sexuality, illness visibility and doctor–patient relationships (Klawiter, 2004; Radley & Bell, 2007).

### Social media visual content, pain, illness and the body

Starting from the early 2000s, digital spaces have become increasingly important for information (Nettleton et al., 2005) and peer support (Burrows et al., 2000) around themes related to health and illness. Social media have been described as inviting new ways of understanding and constructing disclosure and self‐presentation, with positive implications for changing public discourse around traditionally stigmatised health conditions, like HIV (Philpot et al., 2022). Social media self‐disclosure affords social connections built on strong emotional bonds (Petersen et al., 2020), shared experiential knowledge or ascribed expertise in appropriating and distributing biomedical information (Maslen & Lupton, 2019). In fact, connections built on digital platforms have been the focus of recent research into biological forms of citizenship – a citizenship defined by individual but also personalised responsibility in handling health risk and biomedical expertise (e.g. Elton, 2022; Petersen et al., 2019; Petrakaki et al., 2021; Vicari, 2023).

While some attention has been drawn to social media visual representations of illness as enhancing information sharing, peer support and patient advocacy (e.g. Cho et al., 2018; Mazanderani, O’Neill, & Powell, 2013), scholarship has mostly overlooked the role that platforms play in the shaping of these representations. In this sense, the most relevant contribution has probably been advanced by digital media research. Gonzalez‐Polledo and Tarr (2016), for instance, explored how on visual social media (i.e. Flickr and Tumblr) chronic pain is communicated and made understandable for people who do not have first‐person experience of it. Of Flickr images they wrote: ‘they *make pain visible* […]; they appeal to empathy by *making pain felt*’ (Gonzalez‐Polledo & Tarr, 2016, p. 1462, emphasis in original). At the centre of this *making* are self‐portraits, chromatic choices, the use of simile or metaphor, and evocation through image manipulation. The key argument is that social media visual content enables wider distribution of messages that challenge practices of excommunication and ableism. Similar reflections have been advanced in work on Instagram selfies as ‘autopathographic’, that is, as offering first‐person accounts of illness and/or hospitalisation (Tembeck, 2016). Tembeck drew a continuum from the breast cancer projects discussed earlier to this form of social media production, reflecting on the way autopathographic selfies work as ‘acts of coming‐out as being invisibly ill or disabled’ (2016, p. 6). Existing research, for instance, has exposed the role played by ostomy^1^ selfies in nurturing communities of peer support but also in challenging stigma and normalising bodies with ostomy bags within wider publics (Frohlich & Zmyslinski‐Seelig, 2016; Rademacher, 2018). Commenting on Bethany Townsend’s 2014 ostomy selfie originally posted on a Facebook public page and viewed online by over nine million people (BBC News, 2014), Rademacher wrote: ‘the image successfully inserted ostomies into the public discourse, challenges the “regime of shame” associated with non‐normative bodies (Tiidenberg, 2015), and raises awareness about IBD [Inflammatory Bowel Disease] and ostomies among an unintended mass audience’ (2018, p. 3873).

Overall, digital media research has once again foregrounded the role of first‐person visual representations in offering potential to express illness through narrative, voice and biography, which can both provide an entry point into personal ‘worlds of illness' (Radley & Bell, 2007) and challenge societal norms and practices via subversive visualities. This research has however only marginally addressed the role played by the ‘techno‐commercial’ strategies built into social media platforms (van Dijck et al., 2018). For instance, how do these strategies shape the contemporary forms of representation and ‘control’ (Frith & Harcourt, 2007) over the way illness is made visible, narrated and made sense of on social media? In this article, we argue that the situated encounter of platforms’ techno‐commercial strategies and user practices has important implications for the shaping of contemporary visualities of health and illness. We expand on this in the following section.

## CONTRIBUTION: PLATFORM POLITICS, CONTEMPORARY VISUALITIES AND DOMINANT PRACTICES OF HEALTH AND ILLNESS

In line with views that image‐sharing social media reproduce dominant ideologies (e.g. Marwick, 2015; Tiidenberg, 2015), Rademacher suggested that ostomy selfies, while nonnormative, are only likely to gain traction if they meet physical and aesthetic criteria that tend to exclude individuals based on, for instance, race or gender. Therefore, whether social media visual content may offer anchoring potential (Swidler, 2001) for shifts in social practices of health and illness is a question that, we argue, requires reasoning in terms of platform affordances and vernaculars.

Through the lens of affordances, the materiality of a technological object (e.g. a like button) draws broad boundaries around what a user can imagine doing with that object. Platform affordances are therefore key to unpack this work of imagination in the context of contemporary social media (Bucher, 2017; McVeigh‐Schultz & Baym, 2015; Nagy & Neff, 2015; Nau et al., 2022). A key point is that these affordances work in environments that are not neutral (e.g. Ditchfield, 2018): the design of western mainstream social media (e.g. Facebook, Instagram, X) speaks to specific corporate strategies or ‘politics’ (Gillespie, 2010). These media are conceived with a purpose (i.e. to produce corporate profit), with their techno‐commercial design influencing, while not determining, the thriving (or dying) of specific forms of imagination.

Existing research has provided initial evidence of the way the situated interplay between platforms’ techno‐commercial designs and user needs, varies across platforms. In particular, Gibbs and colleagues have explored the emergence of ‘platform vernaculars’ – platform‐specific ‘genres of communication’ with their own ‘styles, grammars, and logics' (Gibbs et al., 2015, p. 257). These evolving vernaculars inform both the way a platform’s techno‐commercial object is designed and the practices developed by its users. A small bulk of cross‐platform research has drawn attention to the implications of platform vernaculars for meaning making, namely for how content is differently *created* and *audienced* within specific social media platforms (e.g. Vicari & Ditchfield, 2024). Part of this research has focused on *visual* vernaculars (e.g. Pearce et al., 2020), drawing attention to platform‐specific visual norms and aesthetics. However, we know little about how these vernaculars nurture the circulation of specific patterns of visuality that may reinforce or hinder dominant practices of ill health and related ‘disease regimes' (Klawiter, 2004; Radley & Bell, 2007).

Drawing on a cross‐platform investigation of popular social media images, in the remainder of the paper we discuss the emergence of specific visualities of hereditary cancer and reflect on how these visualities relate to dominant practices of health and illness.

### Seeing hereditary cancer

The empirical work presented in this article is part of an ongoing Leverhulme Trust‐funded project investigating cancer genetic risk. We focus here on the hereditary cancer syndromes linked to mutations in BRCA genes, which increase the risk of (female) breast, ovarian and, to a lesser extent, other types of cancer, like prostate and male breast cancer (Cancer Research UK, 2024). Upon testing positive for a relevant mutation, carriers are advised to carry out periodic screening to detect cancer in its early stages. Depending on age, family planning and the specific gene mutation, healthy female carriers are offered risk‐reducing surgery, namely, prophylactic mastectomies and (salpingo‐)oophorectomies (the removal of ovaries and fallopian tubes), to prevent cancer (Royal Marsden NHS Foundation Trust, 2021). While being inherited and passed on by both male and female carriers, BRCA mutations have traditionally been associated with (female) breast cancer and primarily addressed as a *woman's* concern. In fact, early research shows that media representations of these mutations have traditionally targeted female audiences. While foregrounding stories centred on family relationships and ‘the horror associated with mastectomy’ (Henderson & Kitzinger, 1999, p. 675), these representations have skimmed over other aspects of the condition and entirely disregarded their impact on male carriers (e.g. on risk of prostate and male breast cancer).

A key aspect of hereditary cancer syndromes is the contested terrain of their visibility: genetic risk is not *bodily* visible, with gene mutation carriers being healthy individuals until their first cancer diagnosis. Visibility is only heightened when cancer prevention or treatment cause bodily changes, namely as a consequence of preventive/therapeutic surgery or invasive treatments (e.g. chemotherapy). This contested visibility also partially contributes to some of the challenges in coping with cancer genetic risk and to the disease regime characterising these conditions. Sociological work shows that unaffected BRCA mutation female carriers often feel they have to defend their choice for or against prophylactic surgery, caught between the imperative of acting responsibly and the anomaly of opting for a medical procedure on their *visibly healthy female* body (Hallowell et al., 2015; Ross Arguedas et al., 2020). In fact, not only do prophylactic surgeries target perfectly functioning body parts; they remove organs that are central to ideas, expectations and embodiments of femininity and womanhood (e.g. Pelters, 2014; Tetteh, 2019). On the one hand, genetic risk measurement legitimises treating healthy bodies as diseased (Finkler, 2010) and therefore eligible to medical surgical intervention or, as Löwy (2010) put it, ‘surgical radicalism’. On the other hand, women who undergo prophylactic surgeries can experience a range of negative emotions related to unexpected bodily sensations, changes in appearance and effects on sexuality (Domchek, 2019; Hallowell et al., 2012). Moreover, the limited biomedical literature on the effects of these surgeries on gender and body image, focuses on reproduction and heterosexuality, reflecting general societal norms (Pelters, 2014). Genetic counselling is commonly framed through normative assumptions about sex and gender (Wellman et al., 2023a), excluding multiple groups in society, like women who want to delay/do not want motherhood, women in same‐sex relationships and trans men.

Existing research shows that carriers of hereditary cancer syndromes turn to social media to journalise and share their experiences (Ross et al., 2018), drawing on varying combinations of lay and expert sources of information (Vicari, 2021a; Wellman et al., 2023b). A common goal of these practices, for instance on Twitter, has been that of educating and supporting through advocacy (Vicari, 2017). The content being shared is likely to vary from platform to platform, with affordances for meaning‐making (e.g. hashtags) enhancing platform‐specific ideational work (Vicari & Ditchfield, 2024). In other words, depending on the platform, certain topics will be foregrounded over others, shaping the way hereditary cancer is discursively constructed (by content creators) and audienced (by all users). But how does the visual shared on social media contribute to this work? How do the BRCA visualities emerging from mainstream social media platforms position themselves against the societal practices of health and illness discussed in this section?

## DATA AND METHODS

To address the questions raised at the end of the previous section, we developed an analysis of popular images of BRCA hereditary cancer syndromes on Facebook, Instagram and Twitter over the course of 12 months. We chose Facebook and Instagram because of their high penetration rates, with the former showing consistent popularity across age groups and ethnicities and the latter being more popular with younger users (e.g. Pew Research Centre, 2024). Twitter, while considerably less popular, has traditionally been a site for professional and news practice (e.g. Burgess & Baym, 2022, p. 8), with its ‘trending’ content often influencing the agenda setting of legacy media. These platforms offer a range of affordances for image sharing and, as shown by work reviewed earlier, they have all proved important for the emergence of discourse and advocacy practices around ill health. We focused on content shared by public accounts, on public pages or on public groups because this is most likely to be seen by both intended and unintended audiences, like was the case for Townsend’s ostomy selfie on Facebook (Rademacher, 2018). In fact, we directed our attention to the *most popular* content because its success at gaining traction is an indicator of enhanced visibility on the platform (e.g. through algorithmic feeds) and of its likelihood to reach wider publics in and outside the platform.

Drawing on Rose’s (2023, p. 47) conceptualisation of the intersecting sites at which meaning is made through visual text (i.e. the site of production, of the image itself, of circulation and of audiencing), we decided to focus *primarily* on the images themselves. This would allow us to provide insight into the way BRCA visualities, that is, how we are made to see BRCA, are constructed through social media images. By focussing on *popular* images, however, our analysis also speaks to circulation and audiencing patterns, namely, it provides insight into platform‐specific dynamics of broadcasting (circulation) and engagement (audiencing). Given our dealing with sets of images, we combined content analysis coding and cultural analytics (Rose, 2023, p. 149).

We collected BRCA‐related English language posts published between 1 May 2022 and 30 April 2023.^2^ These were retrieved through the use of keyword‐based (BRCA, BRCA1, BRCA2) and hashtag‐based (#BRCA; #BRCA1, #BRCA2) queries. We accessed Twitter data via the platform API using TAGS (Hawksey, 2019) and Facebook data via Facebook‐owned CrowdTangle (CrowdTangle Team, 2024). Given the instability of the latter to access Instagram data, for this platform we also relied on the Bright Initiative (Bright Data, 2024).^3^ With the relevant posts for the entire sample period having been collected (5613, 12,916 and 35,844 posts from Facebook, Instagram and Twitter, respectively), we generated monthly datasets. Then, we identified the 10 most popular monthly posts with images: we filtered ‘photo’ posts for Facebook and Twitter and ‘image’ posts for Instagram. We define popularity based on engagement metrics (i.e. reactions count for Facebook, likes count for Instagram and retweets count for Twitter). Finally, using the 4CAT Capture and Analysis Toolkit (Peeters & Hagen, 2022), we downloaded the corresponding visual content^4^: 360 images (10 images × 12 months × 3 platforms).

**FIGURE 1:**
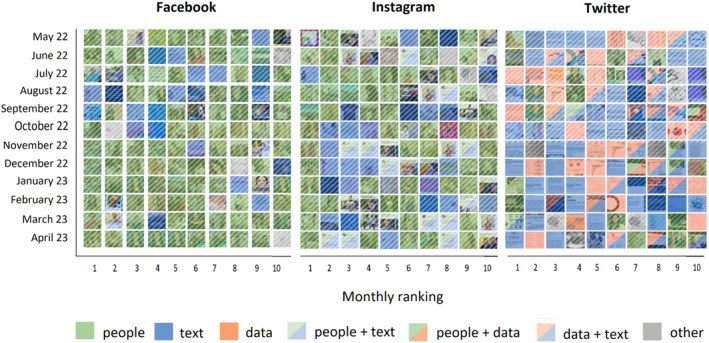
Top 10 monthly BRCA images publicly shared on Facebook, Instagram and Twitter (May 2022–April 2023).

In the first phase of the analysis, we developed a basic coding framework to categorise images based on their content (see Supporting Information S1). Having tested automated image classification tools (i.e. Clarify and Google Cloud Vision API) and assessed their limitations with our dataset, we decided to carry out manual coding instead. Then, we used image grids (Colombo et al., 2023) and colour filtering to visualise and compare the patterns emerging from each platform and gain insight into their visual vernaculars (Figure 1).

In the second phase, we developed a more comprehensive coding framework by combining inductive and deductive codes with codes based on text detection (see Supporting Information S1). For the latter, we used the Google Cloud Vision API to detect words in our dataset (e.g. in image macros or screenshots) and corpus data analysis tool WordSmith to weigh each word’s frequency. Finally, we merged overlapping (inductive, deductive or text detection‐based) codes. While the Phase 1 coding framework was mainly focused on compositional elements, namely on material components (i.e. human elements, words, numbers), phase 2 codes also incorporated technological and social aspects (Rose, 2023, p. 47). For instance, we developed a code to trace the use of screenshots – a *technological* affordance particularly relevant to platform vernaculars (Pearce et al., 2020). But we also coded the presence of selfies as *sociocultural* artefacts at the intersection of dominant ideologies (e.g. Marwick, 2015; Tiidenberg, 2015) and autopathographic representations (Tembeck, 2016). With the coding having been completed, we used visual network analysis (Venturini & Munk, 2021) to identify image clusters in our crossplatform dataset, and explore the emergence of visual genres in the overall mainstream social media ecosystem (of Facebook, Instagram and Twitter). We built a matrix weighing the co‐occurrence of (phase 2) codes in each pair of images and turned this matrix into a network graph (Figure 7). In the graph, nodes (i.e. images) are linked with one another if they share at least one of the phase 2 codes. The topography is rendered in a way that image closeness is based on code sharing: the more codes are shared by a pair of images, the closer these images are in the graph. This process allowed us to identify groups of images with more similarities within the overall dataset, and manually annotate the visual genres that emerge from these groupings.

The project this study originates from received ethics approval from the University of Sheffield in January 2022. All the data analysed in this study were publicly shared, and popular, on social media. However, given their sensitivity and users’ varying expectations of privacy, in the remainder of the article we will use ‘fabrication’ techniques (Markham, 2012): we will present edited images in a way to retain their original message while protecting users’ personal information.

## FINDINGS

### Platform‐specific visual vernaculars

In the first phase of our empirical work, we explored the presence of visual vernaculars. This would allow us to learn about and compare the norms and aesthetics through which BRCA‐related visual content is created and audienced on each platform. The grids in Figure 1 display our study’s images ordered by platform, month of posting and popularity. Colour filters draw on phase 1 codes and highlight patterns of compositionality, in specific terms, each image’s display of ‘people’, ‘text’, ‘data’ or a combination of them (also see Supporting Information S1).

Facebook’s and Instagram’s popular images share similarities: they tend to incorporate human (body) elements and/or text – with the first being slightly more predominant than the latter – and are entirely devoid of numerical expressions. To the contrary, Twitter’s images include text, numerical data and combinations of the two, with a very small number of them picturing people. Vernacular similarity may depend on a number of factors, including the specific ‘politics’ (Gillespie, 2010) or techno‐commercial model, of each platform. For instance, Facebook and Instagram have been owned by the same corporation since 2012 (formerly Facebook, Inc. now Meta). Twitter, as a corporate enterprise, has always distinguished itself from Facebook (e.g. Burgess & Baym, 2022, p. 22) and, at the time of data collection, the platform’s ownership was transitioning to X Corp and experiencing further changes in its techno‐commercial model. Broad convergences or divergences may also depend on the platforms’ specific user demographics or the issue about which content is being created.

**FIGURE 2:**
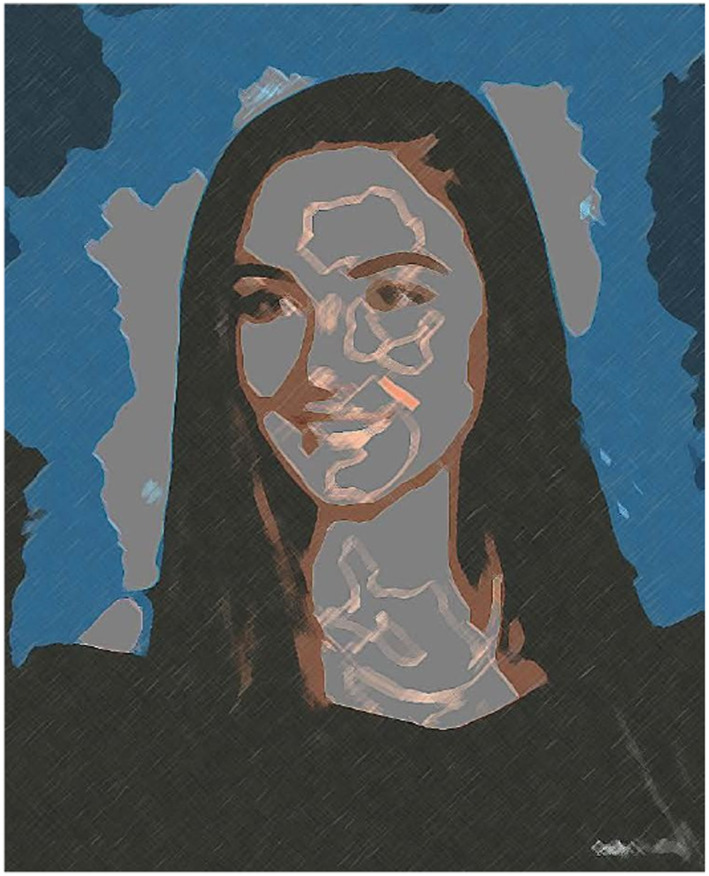
Recurring popular Facebook image (January 2023, rank: 3, 10; April 2023, rank: 3, 4, 5) (edited).

**FIGURE 3:**
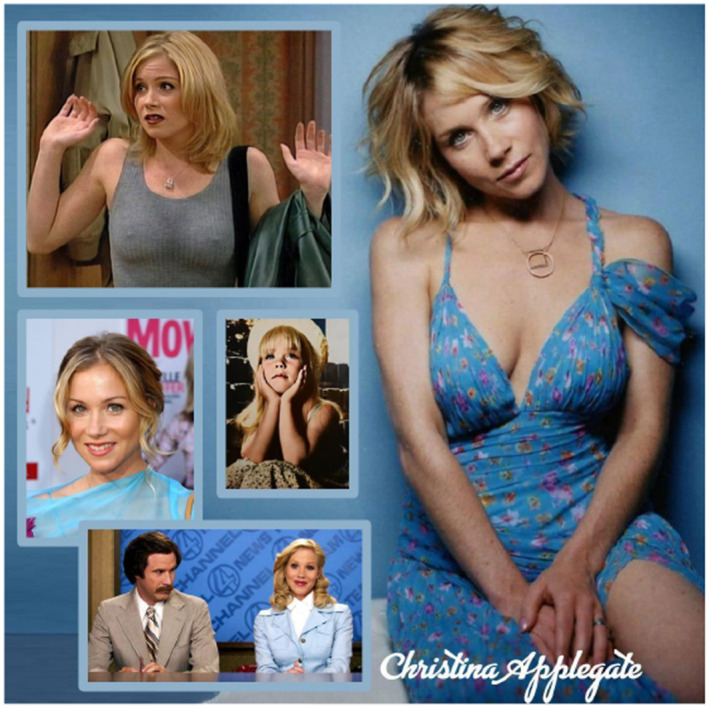
Recurring popular Facebook image (November 2022, rank: 1; December 2022, rank 1; January 2023, rank: 1, 4).

A closer look at Figure 1, however, also suggests more nuanced differences across the three platforms. Facebook’s visual vernacular is more centred than all others on people‐images, with some of these appearing more than once in the grid (e.g. see Figures 2 and 3). The headshot of Figure 2 was included in a first‐person story where substance addiction is framed as a genetic problem, with BRCA being used as a rhetorical device to signify, through simile, genetic predisposition. The five posts incorporating this image – all published by different (‘digital creator’, ‘personal blog’, ‘addiction resources centre’) pages – also share the same or a slightly modified version of the text, with no clear evidence of where the story originated.

The image shown in Figure 3 was included in a long biographical post about actress Christina Applegate, a post that reports on the actress’ BRCA condition and mastectomy experience along with other life events. The post was published four times by the same Facebook ‘Interest page’, ranking as the platform’s most popular BRCA image for 3 months in a row.

The production, circulation and audiencing of the images of Figures 2 and 3 indicate broadcasting mechanisms typical of legacy (especially tabloid) media (Henderson & Kitzinger, 1999). They also suggest a certain popularisation of the BRCA condition, now a symbol of ‘genetic predispositions’ to biological and behavioural dysfunctions (Figure 2), and a noticeable and perhaps relatable event in a celebrity’s life story (Figure 3).

**FIGURE 4:**
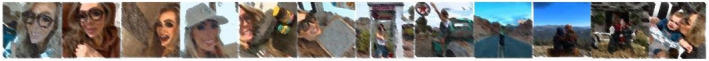
Twelve top Instagram images posted by the same content creator (edited).

On Instagram, repetition does happen, but with personalisation and variation. Figure 4 shows 12 images from the Instagram grid (Figure 1), all having been posted by the same content creator over the course of 8 months (May–December 2022). The corresponding posts are ‘live updates’ of the creator’s ongoing attempts of in vitro fertilisation (IVF) and preimplantation genetic testing (PGT) following the death from cancer of her late husband, a BRCA2 mutation carrier.

**FIGURE 5:**
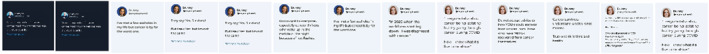
Thirteen popular Instagram images posted by the same content creator (edited).

Similar dynamics of repetition with variation, although through different compositionality, can be found in the 13 images of Figure 5, all also coming from the Instagram grid (Figure 1). They were posted by the same content creator between June 2022 and April 2023, sending inspirational advice on cancer survival and recovery based on first‐person experience (in the first two headshots, the creator is hairless, suggesting chemotherapy treatment).

These examples indicate careful and sustained crafting of personal self‐branding through repetition and aesthetic curation. This crafting is, however, seemingly conducive to different meanings. In Figure 4, technically, ethically but also physically and emotionally demanding complex procedures enter mundane selfies that are devoid of autopathographic expression (Tembeck, 2016): the creator is never shot in hospital settings, with the pictures never representing objects or bodily sensations related to IVF or PGT. Conversely, Figure 5 images constantly engage with cancer‐related themes, often with humourous or sarcastic motivational messages, exclusively drawing attention to the creator’s cancer experience and expertise but limiting any *graphic* expressions of it.

**FIGURE 6:**
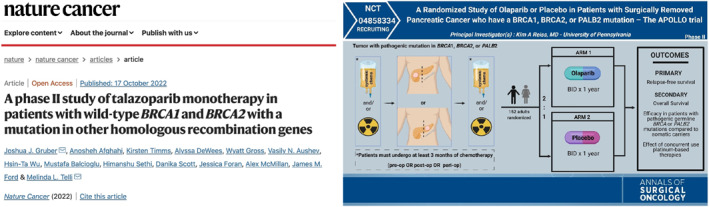
Examples of common popular Twitter image: screenshot of scientific article (left) and poster (right).

Finally, more than a third (43) of the entries from the Twitter’s grid (Figure 1) are screenshots from academic papers or posters (Figure 6), followed by figures and infographics from scientific work and pictures taken at conferences, with only 13 images not explicitly displaying scientific/academic information.

This seems to suggest a strong homogeneity in the production, circulation and audiencing of BRCA content on the platform, with the resulting visual vernacular bounding BRCA as a scientific issue – one for academic networking and self‐promotion.

Having investigated platform‐specific vernacular practices, we will now zoom out and explore how these practices, in their converging and diverging features, lead to the emergence of specific visual genres across the overall social media ecosystem.

### Visual genres across the mainstream social media ecosystem

**FIGURE 7:**
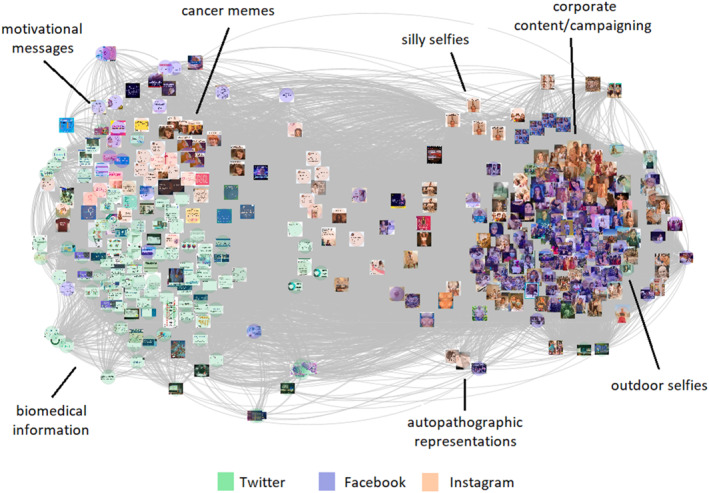
Top 10 monthly BRCA images publicly shared on Facebook, Instagram or Twitter (May 2022–April 2023). Connecting lines indicate shared phase 2 code(s). Layout rendered by ForceAtlas2 algorithm on Gephi 0.9.6 (Jacomy et al., 2014). Manual annotation of emerging visual genres.

In the second phase of our empirical work, we used visual network analysis techniques to inform our interpretive analysis of the visual genres emerging in our cross‐platform dataset. The more (phase 2) codes are shared by any two of the 360 images analysed in the study, the closer these images are in the graph shown in Figure 7. Colour filtering indicates the images’ platform while the manual annotation labels visual genres based on in‐group commonalities.

Figure 7 shows that popular mainstream social media images split almost evenly in two opposite directions: either they picture the body (right) or they explicitly talk about cancer and genes (left). We will further elaborate on this in the next two sections.

#### Picturing the BRCA body

The right side of the graph in Figure 7 is entirely centred on bodily representations that draw, primarily, on Facebook and Instagram vernacular practices with people‐images (see above). A closer look at each cluster allows us to identify distinct visual genres emerging from these practices. While **corporate content** draws on tropes of glamourous femininity typical of pink ribbon campaigning (Hughes & Wyatt, 2015), selfies occupy most of the remaining space. Yet, the BRCA embodiment delivered by these images takes very different forms. **Outdoor selfies** fully exploit the light side of selfie culture, primarily featuring posing white women or couples in likeable outdoor scenes (e.g. see Figure 4). These representations are devoid of autopathographic expression and do not offer meaning that is specific to BRCA as a health condition; without the addition of descriptive hashtags (e.g. #BRCA), these selfies show very little difference from selfies of good health (also see Tembeck, 2016). In other words, they offer nothing new, political or subversive. To the contrary, **autopathographic representations** foreground hospitalisation and bodily experience. This visual genre includes selfies of masked women, sometimes with bandaged chests, lying in hospital beds or wearing head scarves to document chemotherapy treatment, close ups of arms with intravenous (IV) drips, shots of medical devices and pictures of (female) breasts before and after reconstruction. In fact, the white, reconstructed and scarred female breast appears frequently. One image, however, also shows a male individual, bare chested, with scars from their breast cancer surgical treatment, building visibility for a condition that is usually absent from public discourse, that of male breast cancer. The autopathographic expression emerging from these images is nuanced in the way it is constructed by each content creator, playing with different levels of personal and emotional exposure but also with different lenses of spectacularisation (e.g. of the cured, healing or reconstructed body) and normalisation (e.g. of pain). In the context of a well‐established selfie culture of self‐branding and microcelebrity (Marwick, 2015), these images are evidence of the fact that *other* visual expressions sometimes achieve popularity, whether through aesthetic shots of medical devices or very revealing emotional displays. Finally, **silly selfies** do mundane politics (Murru & Vicari, 2021) through self‐disclosure and humour. These images tap into what Hartley (2010) defines as ‘silly citizenship’, playful practices drawing on discursive manifestations of the unexpected. Through humour or irony, they challenge taken for granted cultural practices, in this case of health and illness, and introduce alternative ways to think about the body with, post or expecting cancer. They are *self‐exposing* playful practices, namely they bring together elements from silly citizenship and selfie culture to visualise how a BRCA condition affects the body, for instance, with hair loss due to chemotherapy treatment.

#### Verbalising BRCA cancers and genes

The images on the left side of the graph in Figure 7 tell a different set of stories, primarily built through Instagram and Twitter vernacular practices with text and data visuals (see above). Overall, they centre on cancer and genetics, mostly devoid of explicit forms of embodiment of ill health. **Memes** feature here, again tapping into notions of silly citizenship but without the inclusion of visible self‐exposure. Many of the remaining images in this group are constructed via the use of digital editing tools. This is a relatively recent practice whereby content specifically meant for social media distribution is created via a graphic design app, like the Canva editor app, to fully exploit a platform’s affordances and easily meet its aesthetic norms. These images are particularly suitable to craft and display **motivational messages** on cancer prevention, coping and survival as they allow for easy artistic formatting and background decoration. Some of these messages incorporate a critical element, for instance educating on the way a BRCA mastectomy cannot be compared to a ‘boob job’. By contrasting risk‐reducing surgeries with *aesthetic* surgical practices, this and similar images position these surgeries as *therapeutic* interventions (see also Vicari & Ditchfield, 2024). This positioning legitimises interventionist approaches to cancer risk management, situating personal choice within the cosmology of biological citizenship (e.g. Elton, 2022). Finally, screenshots, which make‐up most of the remaining images on this side of the graph, are mainly used to provide **biomedical information** through cancer and genetics figures, tables, infographics, abstracts or academic posters. This visual genre builds patterns of visuality that are most distant from the personal worlds of illness discussed by Radley and Bell (2007). It indicates a remarkable development in the circulation of scientific work on mainstream social media platforms, especially when compared to what was evidenced in previous research (Vicari, 2017). However, as mentioned earlier, these patterns of visuality tend to be exclusionary, primarily targeting self‐referential and restricted academic and biomedical circles.

## CONCLUSION: ALIGNMENT, LIMINALITY AND (LITTLE) DISRUPTION IN SOCIAL MEDIA IMAGES OF ILL HEALTH

Drawing on Harrison, we argue that social media, as ‘technologies of visual production’, are ‘a crucial determinant of the kinds of imagery produced, the amount that becomes available at particular times, and the kinds of skills required to use them’ (2002, p. 866). However, differently from previous technologies, they work through techno‐commercial mechanisms that function with algorithmic structures of visibility (e.g. based on engagement metrics) and evolve with users’ engagement with and manipulations of these structures. With the increasing importance of the visual in contemporary culture (Rose, 2014), the ubiquity of mainstream social media and their growing influence on the agenda setting of legacy media, call for sociological attention to what and how visual representations of ill health gain popularity, and anchoring potential (Radley & Bell, 2007, p. 369), on these platforms.

Our findings provide evidence of the emergence of platform‐specific visual vernaculars of ill health. While some social media may share general aesthetic norms (e.g. a focus on people‐images), their visual practices will show specificity. Among other factors, platform affordances of privacy and publicity participate in shaping these vernaculars. On Facebook, fully public content is created and shared on ‘pages’ and ‘public groups’, which more closely align with formats typical of legacy (especially tabloid) media. Twitter (now X) and Instagram’s public content is instead commonly created and shared by any accounts, affording more opportunities for first‐person narratives, commodified self‐disclosure and professional self‐promotion/networking. Popular BRCA content on Instagram is often posted by a small number of successful content creators who cultivate and sustain their platform presence via seriality and personalisation. Public disclosure of ill health, whether through specific visual elements or accompanying metadata (e.g. #BRCA), overlaps here with self‐branding and microcelebrity work (Marwick, 2015). On Twitter (now X), popular public BRCA content is created and shared in the context of professional and/or scientific circles, with a seemingly reduced contribution from non‐scientifically minded users.

In sum, each platform shows specific BRCA vernacular practices that are undoubtedly shaped by platforms’ overall politics (Gillespie, 2010), local affordances and user demographics. But how do the visual genres emerging from these practices play with the BRCA ‘disease regime’ (Klawiter, 2004), that is, with the dominant discourses related to the condition? We argue that the BRCA visuality emerging in our study primarily exacerbates existing exclusionary, racialised and gendered practices, while creating some liminal space of imagination and very little disruption.

The prevailing dimension aligns with dominant interpretations of BRCA hereditary cancer syndromes as either a strictly scientific matter or something that concerns a woman’s body. In particular, when it comes to the latter, the BRCA visuality primarily centres on the *white* and *female* breast, *healthy* or *reconstructed*, aligning with normative assumptions about what a woman’s body is often expected to look like and be in western societies. In fact, no image in our dataset offers alternative representations of the female breast, for instance, non‐white and/or following a post‐mastectomy flat closure, like was the case in Metzger and Matuschka pictures, shot in the 70s and 90s, respectively. Not only does this exacerbate current racialised (Happe, 2013) and gendered (Pelters, 2014; Wellman et al., 2023a) practices; but also denies visibility to less known consequences of the BRCA mutations (e.g. ovarian cancer, male breast cancer) and of the possible negative consequences of BRCA‐related preventive or therapeutic interventions (Domchek, 2019; Hallowell et al., 2012).

A second, slightly more subtle, dimension of visuality develops in what we define as a liminal space of imagination; this provides discursive material that hints at renewed, while not necessarily disruptive and certainly not radical ways to think of the BRCA condition. Controlled and extremely curated autopathographic self‐disclosure (Tembeck, 2016), for instance of scars, IV drips or head scarves, offers discursive space to imagine or re‐imagine some of the material elements of the BRCA experience. Like Bethany Townsend’s ostomy selfie, these images embrace the trope of the responsible, beautiful and *likeable* warrior. Any forms of citizenship emerging in this context are highly shaped by the way platformed digital structures (Petrakaki et al., 2021; Vicari, 2021b) and their ‘attention economy’ (Petersen et al., 2019) work and are constrained in the extent to which they challenge exclusionary and biased representations of the ill condition.

The two dimensions discussed so far hardly challenge existing discourses, downscaling optimistic views on the potential of social media practices to prompt social change in the context of existing, normative and exclusionary, disease regimes (e.g. Rademacher, 2018). In fact, only a very small number of images in our study feed into a dimension of visuality that bears more explicit elements of disruption. The photo of a bare chested, scarred individual following a male breast cancer mastectomy is an example of potential to shift public understandings and regimes of shame (Philpot et al., 2022) related to BRCA hereditary cancer syndromes. The same disruptive potential is also at work in the Instagram close‐up of an unsmiling middle‐aged woman, wearing a headscarf and non‐glamorous clothing. An element of visible disruption is also offered by expressions of ‘silly citizenship’ (Hartley, 2010), which offer humourous interpretations of what it means to experience BRCA‐related risk or cancer itself. These images are disruptive because, while normalising ill health, they challenge ableist assumptions of beauty, femininity, and the body. They are, however, as they have always been, a very minor component in the emerging visuality of BRCA hereditary cancer syndromes.

To conclude, granted that the visual has strong influence on understandings and practices of ill health (Harrison, 2002; Radley & Bell, 2007), we argue that attention should be drawn to the specific context in which much of the *contemporary* visual is produced and shared. Mainstream social media platforms show considerable elements of departure from the technological means of visual production discussed in previous work, with important implications for the cultural fabric that they currently afford. Our work points at definite ways in which the techno‐commercial models underlying these platforms shape BRCA visualities or ways to see the condition that very rarely fully depart from dominant practices. It would be interesting to delve deeper into the liminal space we have identified here. For instance, how does social media self‐disclosure intertwine with dynamics of commodification (Mazanderani, Locock, & Powell, 2013) and entrepreneurism? What are the implications for personal and public understandings of health and illness?

Beyond the constraints related to focussing on a one‐language dataset (see note 2), the study presents a few additional limitations that could be addressed in future work. The choice of focussing on still images reduced methodological complexity but also precluded the investigation of reels and stories, which are now a common feature of mainstream social media. The study also excluded the analysis of other mainstream platforms, like TikTok, which may afford more space for non‐normative content and disruptive practices (e.g. Vicari & Ditchfield, 2024).

## AUTHOR CONTRIBUTIONS

**Stefania Vicari**: Conceptualization (lead); funding acquisition (lead); data curation (supporting); methodology (equal); formal analysis (equal); writing—original draft preparation (lead). **Hannah Ditchfield**: Data curation (lead); writing—review & editing (equal). **Yuning Chuang**: Data curation (supporting); formal analysis (equal); methodology (equal); software (lead); visualization (lead).

## CONFLICT OF INTEREST STATEMENT

The authors have no conflict of interest to declare.

## ETHICS STATEMENT

The research has received ethics approval from the University of Sheffield.

## PATIENT CONSENT STATEMENT

Not available.

## PERMISSION TO REPRODUCE MATERIAL FROM OTHER SOURCES

Not available.

## Supporting information

Supporting Information S1

## Data Availability

Due to its sensitive nature, the data analysed in this article have not been made available for future research.

## ENDNOTES

^1^
The ostomy is a surgical opening in the abdomen (stoma) that allows bodily waste to pass into a prosthetic known as an ‘ostomy bag’ (Colostomy UK, 2024).

^2^
We are aware that focussing on one language reduces the significance of our findings. However, this also allowed us to control the quality and relevance of the dataset. Having said that, we are presenting our methodological framework in a way that could be easily replicated with datasets based on other language filters or developing cross‐language investigations.

^3^
The Bright Initiative, powered by Bright Data, offers pro‐bono public data for non‐profit organisations, academic institutions, and public bodies. In February 2024, following a court ruling, Meta dismissed its attempts to prevent Bright Data from scraping data from Facebook and Instagram (Perez, 2024).

^4^
When a post presented multiple images, we only downloaded the ‘cover’ image.
