# Supplementary material for: Contemporary visualities of ill health: On the social (media) construction of disease regimes
Source: Sociol Health Illn. 2024 Sep 20;47(1):e13846. doi: 10.1111/1467-9566.13846 (PMC11684507; doi:10.1111/1467-9566.13846)
Supplement: Supplementary file 1 — Supporting Information S1 [file SHIL-47-0-s001.docx]

# **Supplementary material**

Data File 1: Overall coding framework. The asterisk (*) indicates where text detection contributed to code design.

|  | **Code** | **Value** | **Description** |
| --- | --- | --- | --- |
| **Phase 1**  (Mutually exclusive codes) | Content | People | A person/people is/are in the image |
|  |  | Text | Image containing text |
|  |  | Data | Image containing numerical data. |
|  |  | People & Data | Image containing both ‘People’ and ‘Data’ |
|  |  | People & Text | Image containing both ‘People’ and ‘Text’ |
|  |  | Data & Text | Image containing both ‘Data’ and ‘Text’ |
|  |  | Other | Image not containing any of the above |
| **Phase 2**  (Non- mutually exclusive codes) | Type | Selfie | Self-portrait photograph |
|  |  | Composite | Several images in one |
|  |  | Screenshot | Image that shows the contents of a computer display |
|  |  | Academic article | Image showing article title, authors and/or abstract |
|  |  | Infographic | Infographic(s) |
|  |  | Message | Image showing a message (e.g., inspirational message) |
|  |  | Humour/meme | Meme or humorous image |
|  |  | Before/after | Image displaying something before and after an event (e.g., surgery) |
|  | Setting | Hospital/medical setting | Image with hospital or medical setting |
|  |  | Nature | Image nature setting |
|  |  | Sport | Image with elements of sport activity |
|  |  | Other outdoor | Image with outdoor setting (non nature) |
|  |  | Other Indoor | Image with indoor setting (non medical) |
|  |  | Human in no setting | Image of cut out individuals with no background |
|  |  | Conference | Conference scene |
|  | Topic | Genetics/genomics * | Image about genetics or genomics |
|  |  | Mastectomy * | Image about mastectomy procedure |
|  |  | Giving birth (despite of) | Image about pregnancy despite BRCA condition |
|  |  | Breast reconstruction | Image about breast reconstruction |
|  |  | Sharing a story | Image explicitly displaying some personal information and/or facts |
|  |  | Ovarian cancer * | Image about ovarian cancer |
|  |  | Breast cancer * | Image about breast cancer |
|  |  | Chemotherapy * | Image about chemotherapy |
|  |  | Prostate cancer * | Image about prostate cancer |
|  |  | Pancreatic cancer * | Image about pancreatic cancer |
|  |  | Inhibitor therapy * | Image about any of the following: PARPi, Olaparib, Rucaparib, Talazoparib, Lurbinectedin, inhibitor |
|  |  | Hair regrowth | Image about hair regrowth |
|  |  | Family | Image with family scene |
|  |  | Hug | Image showing a hug |
|  |  | Medical equipment on human body | Image displaying medical equipment on human body |
|  |  | Breast exposure* | Image about breast/showing any form of breast exposure |
|  |  | Science * | Image displaying scientific work, subject (e.g., molecular-looking) |
|  |  | Patients * | Image of individuals as patients |
|  |  | Risk * | Image delivering idea of risk |
|  |  | Mutations * | Image about genetic mutations |
|  |  | Survival * | Image about survival |
|  |  | DNA  * | Image about DNA |
|  |  | Metastatic  * | Image about metastatic cancer |
|  |  | Pathogenic  * | Image mentioning ‘pathogenic’ |
|  |  | Medicine * | Image about medicine |
|  |  | Age * | Image about age/ageing |
|  |  | ASCO  * | Image related to ASCO (American Society of Clinical Oncology) annual conference |
|  |  | USA  * | Image mentioning ‘USA’ |
|  |  | Women  * | Image about/with women |
|  |  | Cell * | Image about/with cells |
|  |  | Health * | Image about health |
|  |  | Syndrome * | Image mentioning ‘Syndrome’ |
|  |  | UK * | Image mentioning ‘UK’ |
